# Incoherent color holography lattice light-sheet for subcellular imaging of dynamic structures

**DOI:** 10.3389/fphot.2023.1096294

**Published:** 2023-02-07

**Authors:** Simon Alford, Christopher Mann, Jonathan Art, Mariana Potcoava

**Affiliations:** 1 Department of Anatomy and Cell Biology, University of Illinois at Chicago, Chicago, IL, United States; 2 Department of Applied Physics and Materials Science, Northern Arizona University, Flagstaff, AZ, United States; 3 Center for Materials Interfaces in Research and Development, Northern Arizona University, Flagstaff, AZ, United States

**Keywords:** Incoherent Color Holography Lattice Light-Sheet, digital holographic microscopy, phase-shifting interferometry, fluorescence microscopy, colocalization

## Abstract

The purpose of the article is to explore the need and advantages of using the incoherent color holography lattice light-sheet (ICHLLS) to provide multiwavelength quantitative monitoring of 3D cellular dynamics in live tissue to further understand complex functions of cells and cellular compartments. We have explored the use of incoherent color holography lattice light-sheet to investigate colocalization of fluorescent markers in live cells in intact tissue. Neuronal structures provide an attractive target for incoherent color holography lattice light-sheet. The cells show a complex architecture in 3D space in which signaling both between cells and within subcellular structures requires colocalization of proteins and lipids to function. During activity and over long periods it is important in understanding these signaling functions in Parkinson’s, Alzheimer’s and motoneuron diseases within live cells in intact tissue. As a proof of concept this article recalls the key aspects in lattice light-sheet imaging and provides a description of the incoherent detection system configuration to actively control dual diffractive lenses phase-shifting at multiple excitation wavelengths sequentially, and per each z-galvo scanning level, with extended field-of-view. The incoherent color holography lattice light-sheet system will allow simultaneous recording of multidimensional object waves that contain intensity in 3D space, phase, and wavelength information. We measure colocalization of fluorescence indicators introduced into live cells in intact neural tissue.

## 1 Introduction

Live cells and particularly neurons are dynamic and three-dimensional in which rapid signaling occurs throughout their complex 3D architecture. Signaling within all cells requires protein-protein interactions and localization of proteins within the framework of lipid and protein structure within the cell (Fivaz and Meyer, 2003). As such understanding brain function requires an understanding of the location and timing of this signaling. A great deal of research has been published examining the spatial relationships between these various structural and signaling elements, but for practical reasons most of this work examines tissue that is fixed and cleared. This improves the visualization of structures and ensures that 3D information can be extracted without time limitations associated with dynamic movement within the cell. This enables relatively slow but accurate point scanning (Denk et al., 1990; Belichenko and Dahlstrom, 1995) or deconvolution approaches to imaging cells. However, fixed tissue loses that very dynamic activity which defines cell function. We seek to develop live cell 3D imaging approaches that can capture multiple wavelengths of fluorescence information in 3D space, sufficiently rapidly to examine dynamic co-localizations between structures and fluorescent molecules. An extreme example of colocalization necessary for function in the central nervous system is in the structural complexity of neurons. To understand neuronal function at all levels, it is necessary to image the spatiotemporal properties of axons, dendrites, and somata in 3-dimensional space simultaneously and with high resolution.

**FIGURE 1:**
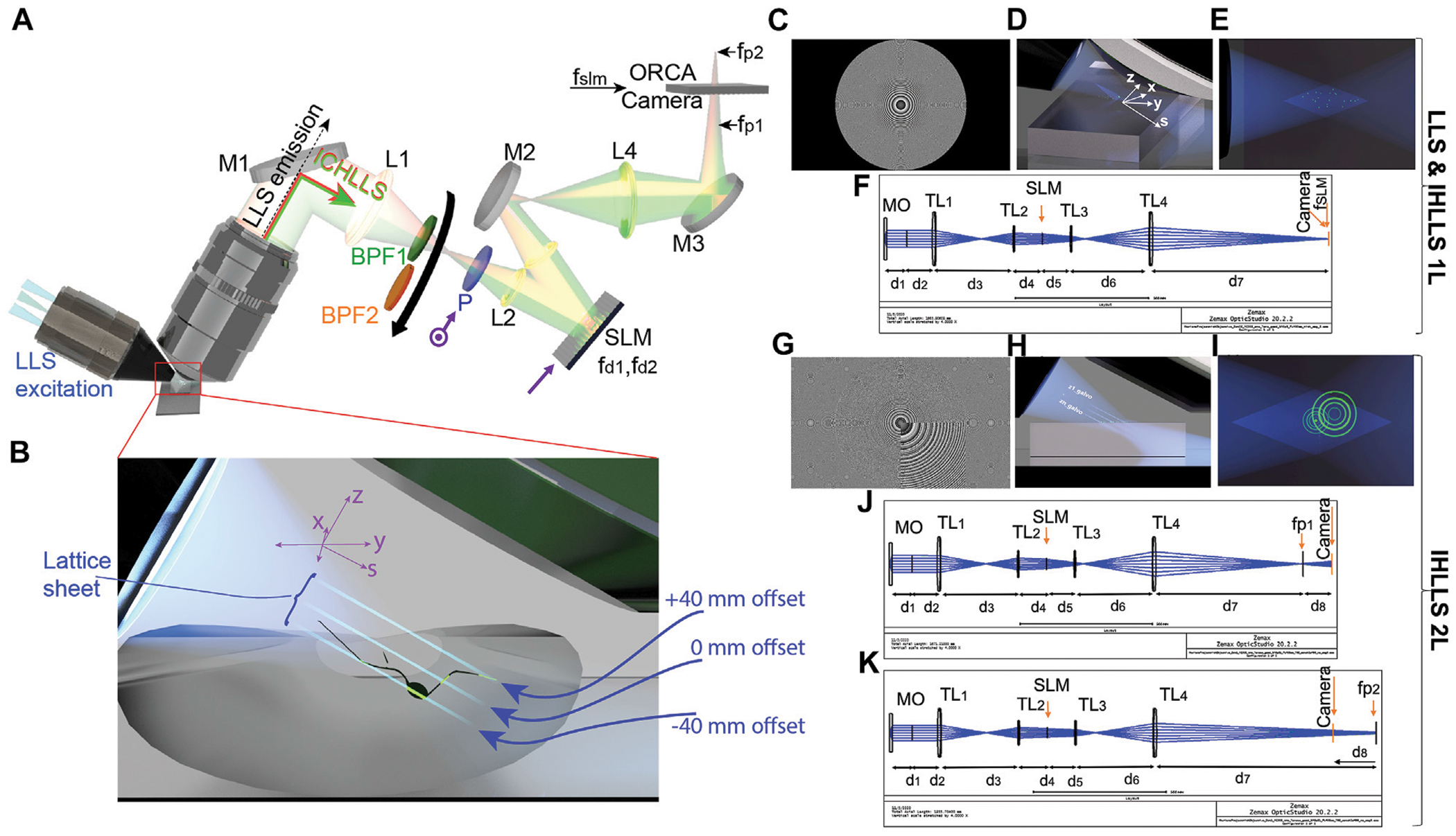
The ICHLLS system. **(A,B)** Schematics of the ICHLLS systems with **(C)** one diffractive lens (ICHLLS 1L) of focal length f_SLM,488nm_ = 400 mm or f_SLM,561nm_ = 415 mm at the phase shift *θ* = 0; **(D)** The axis orientation in the ICHLLS 1L system; **(E)** The top-view of the lattice beams inside the ICHLLS 1L; **(F)** The OpticStudio schematics used to calculate the focal distances for the ICHLLS 1L; **(G)** Schematics of the ICHLLS systems with two diffractive lenses (ICHLLS 2L) with focal lengths f_d1,488nm_ = 220 mm , f_d2,488nm_ = 2356 mm, f_d1,561nm_ = 228 mm, and f_d2,561nm_ = 2444 mm, at the phase shift *θ* = 0, superposed with a slight defocus to bring the objects in focus in the middle of the camera FOV; **(H)** The z-galvo positions in the ICHLLS 2L system; **(I)** The top-view of the lattice beams superposed with Fresnel diffractive lenses inside the ICHLLS 2L; **(J)** The OpticStudio schematics used to calculate the focal distances for the ICHLLS 2L; The system consists of a water immersed microscope objective MO (Nikon ×25, NA1.1, WD 2 mm), lenses L_1_ = L_4_ with focal lengths 175 mm, L_2_ = L_3_ with focal lengths 100 mm; mirrors M_1_, M_2_, M_3_; polarizer P oriented at a 45° angle (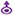) to the active axis of the SLM (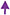); band pass filters BPF_1_ centered at 519 nm (Chroma Tech, 26 nm bandpass width) for the excitation wavelength λ = 488 nm, and BPF_2_ centered at 575 nm (Chroma Tech, 23 nm bandpass width) for the excitation wavelength λ = 561 nm; phase spatial light modulator SLM (Meadowlark Inc.). The light propagates through either pathway LLS emission (dotted black line in A) for the original LLS or pathway ICHLLS (red and green line in A) for ICHLLS, depending on the orientation of sliding mirror. A collimated 30 Bessel beam is focused by an excitation objective lens **(A,B)** which generates a lattice light sheet. While the z-galvo (light sheet) and z-piezo (detection objective) are moved along the z-axis to acquire stacks in LLS and IHLLS1L, **(D,E)**, in IHLLS 2L only the z-galvo is moved at various z positions **(B,H,I)**. For IHLLS, the size of the beam coming out the objective is diminished in half by the relay lens system, L_1_ and L_2_, to fit the size of the SLM. The SLM plane is optically conjugated with the objective back-focal-plane. The diffraction mask in the original LLS system was positioned for all experiments on the anulus of 0.55 outer NA and 0.48 inner NA. The CMOS camera, tube lens, filter, and detection objective lens are used for fluorescence detection. The detection magnification *is* 62.5. The width of the light sheet in the center of the FOV is about 400 nm. *x*-axis is the direction of the x-galvo mirror motion, *z*-axis is the direction of the z-piezo mirror motion, and s-axis is the direction of excitation light propagation. For values of distances d1 to d8, see Supplementary Figure S1.

Lattice light-sheet (LLS) imaging helps to resolve dynamical changes in 3D (Tahara et al., 2020), but in common with all 3D imaging the need to maintain the emission objective in focus with the object causes mechanical distortion and takes time. 3D information is obtained by scanning the excitation lattice sheet in the z-dimension using a z-galvanometric mirror (z-galvo) in the excitation light path after the creation of the Bessel beams. The z-scanning principle in LLS, is that both this z-galvo and the detection objective (z-piezo), synchronize in motion to scan the sample in 3D, Figure 1B. Furthermore, because the LLS system excites with a lattice with regions of positive and negative interference, the resulting image intensity will vary. This variance is smoothed by dithering the beam in the x plane with a similar x-galvo. While LLS beams are coherent and produce stronger modulation depth than incoherent patterned light sheets, they work by causing fluorescence in the sample which results in emission light that is incoherent. We explore here the self-interference property of this emitted fluorescent light, at multiple wavelengths, in which interference patterns are created to generate holograms of a 3D object with the depth information encoded in these patterns.

Incoherent color digital holography has been an active area of research and development for applications in fluorescence microscopy. Multicolor incoherent digital holography system with a white-light source using a monochrome image sensor and a wavelength-dependent phase-modulation array has been applied to incoherent-light hologram sensing (Tahara et al., 2020). Full-color quantitative phase imaging of HE-stained mouse kidney cells using a computational coherent superposition (CCS) has also been demonstrated (Tahara et al., 2021). Super-resolution imaging with full-color incoherent imaging using Fresnel incoherent correlation holography (FINCH) was achieved (Rosen and Brooker, 2007a; Rosen et al., 2021).

We built a single-wavelength incoherent holography lattice light-sheet (IHLLS) (Potcoava et al., 2021a; Potcoava et al., 2021b; Rosen et al., 2021), at 488 nm, but here we combine IHLLS with multiwavelength information at 488 nm and 561 nm, termed incoherent color holography lattice light-sheet (ICHLLS), to provide quantitative monitoring of 3D cellular dynamics in live tissue. The system could be developed further for all four excitation laser lines (405, 488, 561, and 642 nm) of the existing LLS system by configuring a phase spatial light modulator placed in the incoherent detection arm of the instrument to actively control dual diffractive lens phase-shifting at all colors sequentially. The ICHLLS system is built as a new detection module on an existing LLS system.

This holographic technique gives us the possibility to access the complex amplitude of the object’s wavefront, which describes both the amplitude and phase of a light wave. Amplitude and phase measurements acquired with our new instrument provide intrinsic instrumental simplicity, larger scanning area, larger z-galvanometric mirror range, and higher resolution when compared to the original LLS schemes. Phase imaging provides quantitative information on the state and size of subcellular structures.

In this paper we focus on assessing the colocalization between dual-color excited structures and fluorescent molecules detected in incoherent holographic images, recorded using ICHLLS. The rest of the paper is structured as follows: in the next section the principle of the ICHLLS system is provided with a short theory on the colocalization approach. Section 3 covers the materials and methods. Section 4 covers a series of ICHLLS experiments using calibration beads and neuronal cells. Finally, Section 5 discussions and conclusion.

## 2 Principle

### 2.1 Instrument presentation and optical design

Various aspects of IHLLS system at only one excitation wavelength of 488 nm have been described in (Potcoava et al., 2021a; Potcoava et al., 2021b), Figures 1A, B, including the IHLLS with one (IHLLS—1L) lens, Figures 1C–F or two diffractive lenses (IHLLS-2L), Figures 1G–K. The imaging detection in ICHLLS still uses the Fresnel incoherent correlation holography (FINCH) (Rosen and Brooker, 2007b; Rosen et al., 2011; Katz et al., 2012; Rosen and Brooken, 2012; Siegel et al., 2012; Rosen and Kelner, 2014; Rosen et al., 2021) principle, but at two excitation wavelength, 488 nm and 561 nm. The emitted fluorescent light from a 3D object is split into two spherical beams with different radii of curvature by a phase spatial light modulator (SLM) to create a single channel on-axis interferometer, Figure 1A. The interference patterns created by the two beams at each excitation wavelength create Fresnel holograms. The beam splitter of an interferometer is replaced, in our case, by the phase SLM (Meadowlark Inc.; 1920 × 1,152 pixels, pixel pitch 6.5 μm). The SLM transparency for the two beams has the expression:

(1) $$\left[{\text{C}}_{1}\text{Q}\left(-\frac{1}{{\text{f}}_{\text{d}1,\lambda }}\right)+{\text{C}}_{2}\;\text{exp}\;\left(\text{i}\theta \right)\text{Q}\left(-\frac{1}{{\text{f}}_{\text{d}2,\lambda }}\right)\right]$$

where $\text{Q}\left(\text{b}\right)=\exp\left[{{\text{i}\pi \text{b}\lambda }_{1,2}}^{-1}\left({\text{x}}^{2}+{\text{y}}^{2}\right)\right]$ is a quadratic phase function, C_1,2_ constants, f_d1,λ_, f_d2,λ_ are the two diffractive lenses focal lengths for the two excitation wavelengths, and θ is the shift phase factor of the SLM. Only one component of the electric field vector is affected by the SLM as a wave that is modulated with one of the two diffractive lenses, and the other component as a wave without modulation. This is possible by using a polarizer P oriented at a 45° angle to the active axis of the SLM and two diffractive lenses uploaded on the SLM with 50/50 randomly selected pixels, Figure 1A. The two diffractive lenses focus on the planes f_p1_ and f_p2_, in the front and behind the camera.

When f_d1,λ_ = ∞, Figure 1C with an uneven distribution of the two constants, in front of each term in Eq. (1), with only one the phase factor of θ = 0, the expression becomes:

(2) $$\left[0.1+0.9\;\exp\;\left(\text{i}\theta \right)\text{Q}\left(-\frac{1}{{\text{f}}_{\text{SLM},\lambda }}\right)\right]$$

This case refers to the technique called ICHLLS 1L and it is used for calibration purposes. The z-scanning principle in ICHLLS 1L, same as in LLS, is that both the z-galvanometric mirror (z-galvo) and the detection objective (z-piezo), synchronize in motion to scan the sample in 3D, Figures 1D, E, but the axial resolution could be lower than the axial resolution in LLS due to the blurring effect of the constant phase lens added on the SLM that focusses to infinity.

In ICHLLS 2L technique, C_1,2_ = 0.5 and the phase factor has four phase shifts, θ = 0, π/2, π, 3π/2. In this case the two diffractive lenses of finite focal lengths, with non-shared randomly selected pixels, Figure 1G, are simultaneously uploaded on the SLM and four phase-shifting intensity images with different phase factor are recorded and saved in the computer sequentially and numerically processed by in-house diffraction software. The detection objective is also kept fixed, but the z-galvanometric mirror moves to reach various depths in the sample, Figure 1H, and to increase the visibility of the Fresnel patterns, Figure 1I. The ICHLLS 2L technique is used for the actual 3D sample imaging.

The physical distances between each sequential optical component in Figure 1A, and the focal distances for the two excitation wavelengths were calculated using the OpticStudio (Zemax, LLC). First, we calculated the focal distances for the ICHLLS 1L, Figure 1F, for both excitation wavelength, with the condition that the transversal magnification in the new system has to match the transversal LLS magnification of 62.5. The focal lengths are f_SLM,488 nm_ = 400 mm and f_SLM,561nm_ = 415 mm. After that, we designed a multi-configuration optical system, IHLLS 2L, Figures 1J, K, with the condition that the height of the two beams for each excitation wavelength, generated by the two lenses, was equal in size at the camera plane for a perfect overlap. In this case the focal distances of the two diffractive lenses for each excitation wavelength are f_d1,488 nm_ = 220 mm , f_d2,488 nm_ = 2356 mm, f_d1,561 nm_ = 228 mm , and f_d2,561 nm_ = 2444 mm.

**FIGURE 2:**
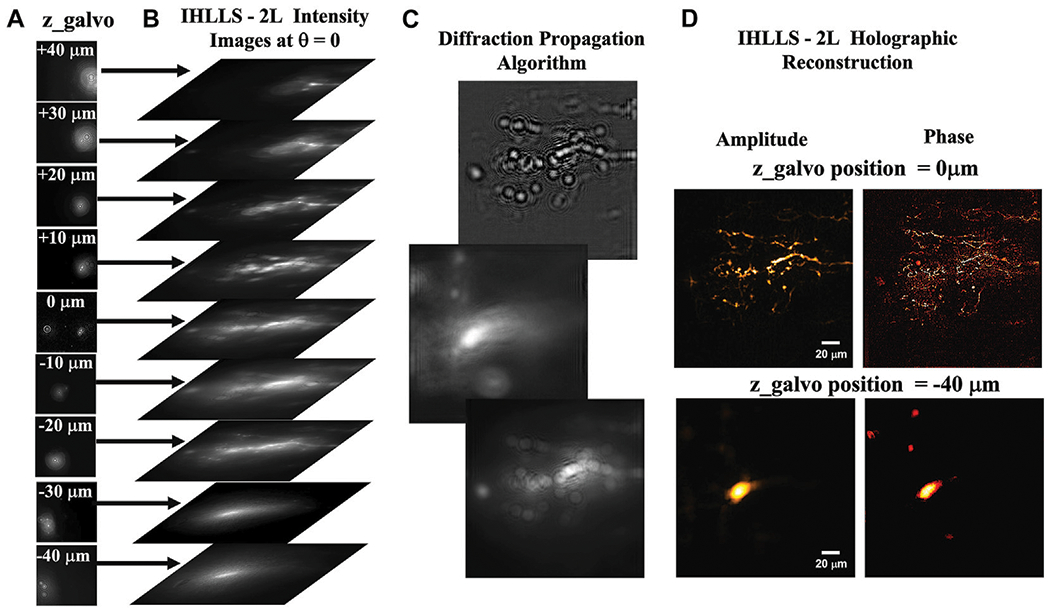
The ICHLLS system diffraction. **(A)** The 9 z-galvo position hologram intensity images: z_galvo_ = ± 40μm± 30μm; ± 20 μm; ± 10 μm,; 0 μm; **(B)** Hologram intensity images at θ = 0, for each z-galvo level; **(C)** Example of hologram reconstruction at different z-galvo positions; **(D)** The reconstructed amplitude and phase at two z-galvo positions; z_galvo_ = −40 μm and 0μm. The FOV is 208 μm^2^ × 208 μm^2^ and pixel size 0.101 μm.

In both ICHLLS techniques, ICHLLS 1L and ICHLLS 2L, the two wavefronts, created by the two diffractive lenses uploaded on the SLM, interfere with each other at the camera plane to create Fresnel holograms. In ICHLLS 1L the 3D scan is achieved by moving simultaneously the z-galvo mirror and z-piezo objective controller for a range of 3 0 μm, in *δz*_LLS_ = 0.101 μm increments through the focal plane of the detection objective, for a phase shift on the SLM of θ = 0 . A bigger scanning volume with a maximum scanning area of 208 × 208 μm^2^ is achieved by using ICHLLS 2L where only the z-galvo was moved within the Δ*z*_galvo_ = ± 40 μm displacement range, above and below the reference focus position of the objective (which corresponds to the middle of the camera FOV), at 9 z-galvo positions, Figure 2A; *z*_galvo_ = ± 40 μm ± 30 μm; ± 20 μm; ± 10 μm; 0 μm. We selected a small number of z-galvo positions symmetrically distributed above and below the lattice plane, to cover the maximum FOV of the camera, and to show that the 3D objects could be reconstructed with fewer z-galvo steps than the conventional LLS or it is corresponding incoherent version ICHLLS 1L. In a practical application of this system, we will increase the number of z-galvo steps and speed up their acquisition using software solutions. Four interference patterns were created for each z-galvo position and excitation wavelength using a phase shifting technique (θ = 0, θ = π/2; θ = π, θ = 3*π*/2), Figure 2B, and further combined mathematically to obtain the complex amplitude of the object point at the camera plane, U(u, v). Then, the complex hologram is propagated, Figure 2C, and reconstructed at the best focal plane using a custom diffraction Angular Spectrum method (ASM) (Kim, 2010) routine programmed in MATLAB (MathWorks, Inc.).


(3) $$\text{U}\left(\text{u},\text{v}\right)=\text{A}\left(\text{u},\text{v}\right)\;\text{exp}\;\left(\text{i}\phi \left(\text{u},\text{v}\right)\right)=\frac{1}{4}\{({\text{I}}_{\text{H}\left(\text{u},\text{v},0\right)}-{\text{I}}_{\text{H}(\text{u},\text{v},\pi )})+\text{i}\left({\text{I}}_{\text{H}\left(\text{u},\text{v},\pi /2\right)}-{\text{I}}_{\text{H}\left(\text{u},\text{v},3\pi /2\right)}\right)\}$$

where: *A*(*u*, *v*) is the amplitude of the image, Figure 2D left panel, I_H_ are the hologram intensities, Figure 2B, and phase, Figure 2D right panel:

(4) $$\emptyset \left(u,v\right)=arctan\left[\frac{I_{H}\left(u,v,\pi /2\right)-I_{H}\left(u,v,3\pi /2\right)}{I_{H}\left(u,v,0\right)-I_{H}\left(u,v,\pi \right)}\right]$$

The depth of the object points is encoded by the density of the rings in the Fresnel holograms, meaning that each plane in the image space reconstructed from the Fresnel hologram is in focus at a different axial distance. In this manuscript the phase data represent the object phase retrieved at certain axial position of z-galvo mirror. We intend to include extra variables for future work to obtain biomedically relevant information on the state of cells.

The spatio-temporal performance of a single-wavelength incoherent holography lattice light-sheet was already demonstrated in our previous work (Potcoava et al., 2021b). The technology provides the imaging resolution comparable or better than the conventional LLS’s resolution in dithering mode and with maximum detector FOV. The LLS resolution in the *z*-axis is limited by the light sheet illumination depth of about 400 nm, but LLS microscopy enables diffraction limited resolution in the x and y. The axial resolution in IHLLS 2L and ICHLLS 2L can be improved by recording and reconstructing images at multiple z-galvo steps and minimizing the hologram reconstruction increment, δz, to achieve better localization of the sample points.

### 2.2 Automatic colocalization

Pearson’s correlation coefficient (PCC), Manders’ overlap coefficient (MOC), and Manders’ colocalization coefficients (M1, M2) are all generally accepted methods to assess the spatial overlap of multi fluorescent labels recorded at different emission wavelengths when imaged at a particular spatial resolution (Manders et al., 1992; Manders et al., 1993; Costes et al., 2004; Li et al., 2004). These coefficients underline two aspects of the co-localization: correlation (PCC) and co-occurrence (MOC, M1, M2).

The correlation refers to the linear relationship of the pixel intensities in the spectral channels or what percentage of variability in one channel is caused by variability in the other channel, and the co-occurrence represents the degree of channel overlap (Adler et al., 2008). PCC can range from −1 to 1, with PCC = 1 meaning perfect positive correlation, PCC = −1 meaning perfect negative correlation or anticorrelation, and PCC = 0 no correlation. MOC ranges between 0 and 1 and it represent the percentage of the overlapped pixels in the spectral channels. The correlation-based interpretation of the MOC is that a value of 0 corresponds to negative correlations, 0.5 to no correlation, and 1 to positive correlation (Cordelieres and Bolte, 2014). The MOC was described as representing the true degree of colocalization with values from 0.6 to 1 values indicating positive colocalization (Zinchuk and Grossenbacher-Zinchuk, 2009).

The two MOC coefficients, M_1_ and M_2_, represent the fraction of co-localizing objects in each component of a dual-spectral channel image above threshold intensity values. As an example, for our excitation wavelengths, 488 nm and 560 nm, M_1_ is the percentage of pixels above threshold pixels in image recorded at 488 nm that overlap above threshold pixels in image recorded at 561 nm and M_2_ is the percentage of pixels above threshold pixels in image recorded at 561 nm that overlap above threshold pixels in image recorded at 488 nm.

This method is not intended for analyzing molecular interaction, which requires super-resolution techniques such as electron (Pastorek et al., 2016) or fluorescence resonance energy transfer microscopy (Day et al., 2001), but are useful first step to show that our IHLLS technique is capable of imaging biological samples in multiple spectral channels.

## 3 Materials and methods

### 3.1 Sample preparation

Fluorescent latex beads of 500 nm (λ_exc_ = 488 nm, λ_em_ ~ 520 nm), F-8888, and 1 μm (λ_exc_ = 561 nm, λ_em_ ~ 575 nm), F-8820, (ThermoFisher Scientific, United States) were used as test objects. The bead solution (2% solids) was diluted 1:4000 with distilled water and briefly centrifuged in a desktop centrifuge for 1 min. Clean coverslips were prepared by applying 1 μL as a thin layer that was left to dry. After drying, the cover slip was mounted in the sample holder under distilled water.

Lamprey motoneurons were imaged in live intact isolated spinal cords. Of larval lampreys (*Petromyzon marinus*). Twenty-four hours prior to isolation of the spinal cord, the animal was anesthetized with tricaine methanesulfonate (MS-222; 100 mg/L; Sigma, St. Louis, MO) in pH buffered cold saline. Under anesthesia the myotomal muscle wall (1–3 segments) was injected with 2 μL of 10 mM 10,000 MW dextran conjugated to each of Alexa 488 and Alexa 555. The animal was allowed to recover and maintained in an isolated aquarium at 10C for 24 h. The animals were again anesthetized with (MS-222; 100 mg/L), decapitated, and dissected in a cold saline solution (Ringer) of the following composition (in mM): 100 NaCl, 2.1 KCl, 2.6 CaCl_2_, 1.8 MgCl_2_ or 1.8 MgSO_4_, 4 glucose, 5 HEPES, adjusted to a pH of 7.60 with NaOH and to a final osmolarity of 270 ± 5 mOsm.

The spinal cord was isolated and placed ventral side up to expose the motoneurons closest to the imaging system in a cooled, small-volume chamber with a sylgard floor. The chamber and spinal cord were then transferred onto the customized stage of the LLS microscope. The recording chamber was continually superfused with cold, oxygenated Ringer (8°C–10°C) for the duration of the experiment while maintained under the dual lenses of the LLS system. The myotomal injection of dye allowed retrograde transport of dye throughout the axons, somata and dendritic fields of motoneurons in the spinal ventral horn, with labeling with both Alexa 488 and Alexa 555, allowing a demonstration of colocalized dye imaging of neurons in a complex 3D space.

### 3.2 Image acquisition and processing

The entire system was controlled by the original LLS software based on LabView platform (National Instruments) with the diffractive SLM (Meadowlark Inc.) synchronized with the ORCA camera for the IHLLS module. The diffractive lenses with different phase factor were generated in MATLAB (MathWorks, Inc.) for each of the two emission wavelengths, 520 nm and 575 nm.

Data acquisition is performed in two steps: a) move the z-galvo mirror to a specific z-galvo position (offset) and record four phase-shift holographic intensity images per z-galvo position, at z-galvo: ± 40, ± 30; ± 20; 0 μm ; b) switch the excitation wavelength and perform step a) for the same offset z-galvo positions. The image acquisition time for recording an intensity image is 50 ms in the middle of the FOV or at z-galvo position of 0 μm , increasing progressively to 100 ms at edges of the z-galvo mirror, ± 40 μm , per each diffraction pattern.

Usually, image processing steps are the same for most of the diffraction techniques. Each hologram processing can be sub-divided as follows: 1) Image pre-processing in which a background correction is performed by subtracting an average background level obtained by measuring the mean intensity of each stain outside the cells; 2) Hologram reconstruction in which the complex hologram is propagated and reconstructed at the best focal plane using a custom diffraction method routine in MATLAB (MathWorks, Inc.); and 3) Object feature extraction performed on the reconstructed amplitude and phase images using FIJI (ImageJ).

## 4 Experiment and results

### 4.1 Calibration

**FIGURE 3:**
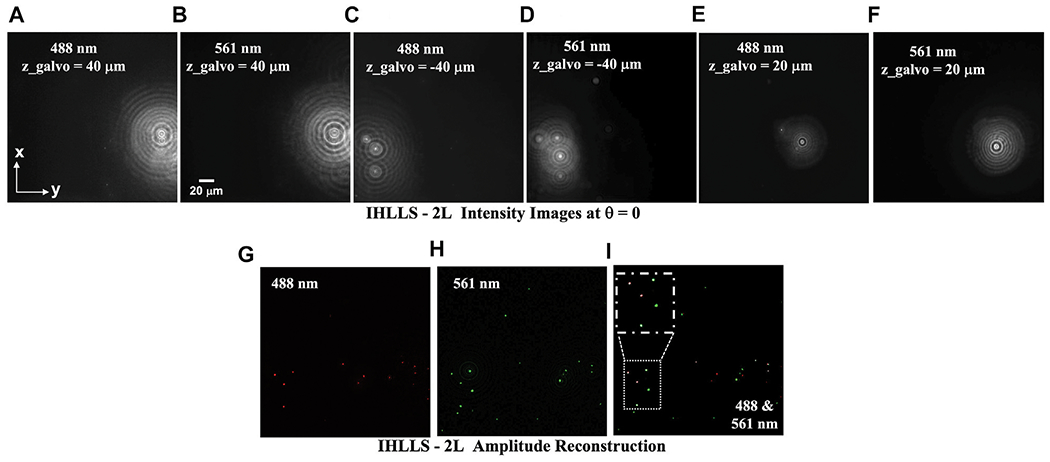
IHLLS 2L beads volume reconstruction of a mixture of fluorescent beads; **(A,C,E,G)** 500 nm (488 nm) diameter fluorescent beads, and **(B,D,F,H)** 1 μm (561 nm) diameter fluorescent beads z-galvo levels ± 40; 20 μm are displayed, at the phase-shift *θ* = 0; The xy views of beads after the hologram reconstruction, at **(G)** 488 nm excitation, **(H)** 561 nm excitation, represent the z-max projection of all the best z-reconstructed planes; and **(I)** The colocalization map of the *xy* planes in **(G)** and **(H)**. The FOV is 208 μm^2^ × 208 μm^2^ and pixel size 0.101 μm.

As a proof-of-concept, we performed color fluorescence imaging using conventional ICHLLS 1L then ICHLLS 2L in which we exploited coherent and incoherent holographic imaging at two excitation wavelengths, 488 nm and 561 nm. To calibrate the system, we demonstrated color detection of a mixture of 500 nm (488 nm) Figures 3A, C, E, G and 1 μm (561 nm) diameter fluorescent beads, Figures 3B, D, F, H. Emission during 488 nm excitation is centered at 520 nm and following 561 nm excitation is centered at 575 nm. Using the IHLLS 2L, we recorded four phase-shift images per z-galvo position at z-galvo ± 40, ± 30, ± 20, 0 μm, for both excitation wavelengths, reconstructed the best z position and combined all the z-reconstructed planes. Examples of holograms recorded at z-galvo levels ± 40, and 20 μm are displayed in Figures 3A–F for the phase shift θ = 0. Reaching certain z-galvo levels is possible by combining the two diffractive lenses of focal lengths, f_d1,488 *nm*_ = 220 mm, f_d2,488 *nm*_ = 2356 mm, f_d1,561 *nm*_ = 228 mm, and f_d2,561 *nm*_ = 2444 mm.

When imaging individual beads with both wavelengths, shorter wavelength excitation, 488 nm, bleeds through when imaging 561 nm beads, Figure 3I. The superposition of the z-max projections of selected z-reconstructed planes for each of the two laser beams are shown in Figure 3G, H and the colocalization map of the beads at both wavelengths is shown in Figure 3I. We zoomed in an area of the colocalization map to show the 488 nm beads are superposed with the 561 nm beads, these beads are colored in white.

IHLLS phase images contain the depth dependent phase information derived from the IHLLS holograms and the reconstructed IHLLS images display the complex holograms propagated to the best focal plane. The max projections of the reconstructed volume of the 1 μm beads sample were obtained by changing the z-galvo levels to ±40 μm, ±30 μm, ±20 μm, ±10 μm, and 0 μm, Figures 3A–F. Thus, color 3D sensing of multiple beads and cells was performed.

### 4.2 Imaging neurons

**FIGURE 4:**
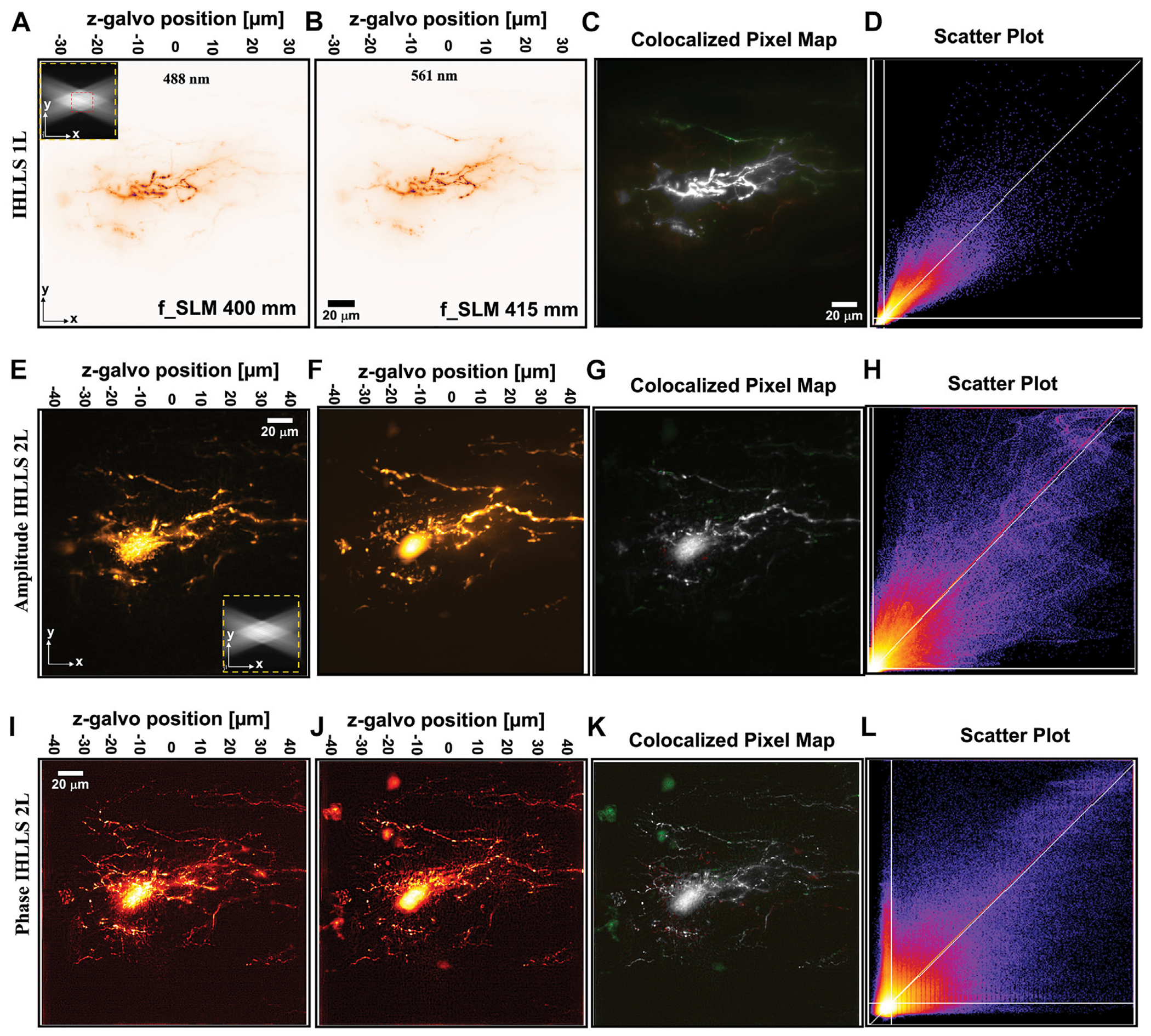
IHLLS-2L colocalization of two colors of dye Alexa 488 nm and 561 nm throughout the complex 3D space of the motoneuron dendritic trees; The *xy* volume map projection, FOV 208 μm^2^ × 208 μm^2^, in an ICHLLS 1L with only one diffractive lens of focal length 400 mm for 488 nm **(A)** and 415 mm for 561 nm **(B)**, without deconvolution; The xy projections were built from 400 *z*-axis planes in the range −30μm to 30μm in 0.101μm steps. The excitation Bessel beams are displayed in the upper-left corner of **(A)** to show the orientation of the beams (FOV 208 μm^2^ in yellow for the ICHLLS and FOV 52 μm^2^ in red for the original LLS). The *xy* volume map projection from ICHLLS −2L reconstructed amplitude images, FOV 208 μm^2^ × 208 μm^2^, and the corresponding phase images, with only 9 *z*-axis planes in the range −40 μm to 40 μm in 10 μm steps, exciting sequentially with 488 nm **(E,I)** and 561 nm **(F,J)**; The colocalization maps **(C,G,K)** and the scatter plot **(D,H,L)** for each case are displaced on the right.

To examine the effects of applying ICHLLS holography, neurons fluorescently labeled live *in situ* in the central nervous system (lamprey spinal ventral horn neurons), Figure 4, were used as test samples. Fluorescent dyes, Alexa Fluor^™^ 488 nm and 568 nm hydrazide, were injected by microinjection into the neuronal lamprey cell.

A goal of this study is to enable colocalization or separation of probes of separable fluorescent wavelength to be identified in the 3D structure of neurons during live cell recording in intact tissue *in situ*. Such recording presents challenges. Photodamage from excessive excitation will destroy the neurons, the tissue is dynamic and can move during extended periods of recording, and the tissue is complex and diffractive limiting the lattice beam penetration depth as well as the clarity of the emitted light.

To examine the effects of applying ICHLLS holography, neurons were fluorescently labeled live *in situ* in the central nervous system. We used well-characterized neurons in the intact spinal cord as a test example (Wallen et al., 1988). The lamprey spinal cord can be removed intact from the animal and maintained alive, *in situ* for up to 48 h without noticeable ultrastructural damage. The tissue used from larval animals (*Petromyzon marinus*) was approximately 200 μm deep and 750 μm wide. Within this structure motoneurons occupy most of half the spinal cord, projecting laterally and 350 μm and from the ventral surface of the spinal cord to a depth of approximately 80 μm. The dendritic tree is complex and receives input from ventromedial (left of images) as well as lateral tracts (right side of images). The Soma is in the ventral horn and approximately 60–80 μm in the *z*-axis from the ventral surface (Buchanan et al., 2008). Therefore, to use as test samples, motoneurons were labeled as described in the methods section to cause the colocalization of two colors of dye Alexa 488 nm and 555 throughout the complex 3D space of the motoneuron dendritic trees, Figure 4. An example of a 3D LLS scan of the two colors is shown, Figure 4A, B in which the entire field of the camera (208 μm^2^ × 208 μm^2^) and depth of the scan (300 planes at 0.1 μm intervals) is shown by averaging all z-planes.

The first step in showing that the location of neurons in dual-channels are closely related is to overlay the dual-channel data, 488 nm in red and 561 nm in green, and then to state that anything that shows up as white shows colocalisation. The second step in the colocalization process is to use a scatter plot diagram of the pixel values of the dual-channel images against each other, to calculate the colocalization parameters. The data information of a given pixel in the 488 nm image is used as the *y*-coordinate of the scatter plot and the data information of the corresponding pixel in the 561 nm image as the x-coordinate. A pixel distribution along a straight line and a Pearson’s or Mander’s coefficients close to 1 means that both channel distributions are linked and colocalized.

The imaged neuron was labeled with dyes attached to identical molecular weight dextrans that are expected to label the neurons to the same extent. This is confirmed by imaging with IHLLS-1L similar to scanning with the original LLS, Figure 4(A–D) in which correlation and colocalization coefficients are high (Table 1). Degree of colocalization of the two dyes using IHLLS 1L is shown in green/red overlay, Figure 4C, and the scatter plot map in Figure 4D. The higher the MOC, the stronger M1, and M2 are, and the evidence for colocalization is high. Comparison between the IHLLS-1L reconstruction and from both amplitude and phase reconstructions using IHLLS-2L demonstrates that the IHLLS-2L approach enables accurate colocalized structures. Similar coefficients of colocalization are recorded from IHLLS-2L amplitude data, Figure 4(E–H). Colocalization coefficients in the phase data from IHLLS-2L demonstrates less colocalization. This reflects the very high sensitivity of this approach to z-plane offset and the fact that in live tissue small movements occur throughout the recording. The IHLLS-2L data, both amplitude and phase, also demonstrates the capability of this approach to detect faint data in the periphery of the recording. This indicates the possibility of extracting data from larger volumes of 3D structure.

## 5 Discussion and conclusion

We have demonstrated that 3D amplitude and phase information can be extracted from fluorescent samples scanned using a lattice light-sheet, and that this can be achieved at two or more wavelengths. This also enables an increase in resolution compared to the original LLS approach of moving the sample or the objective lens. For proof of principle, we have demonstrated that this can be achieved with 9 z-galvo positions of the ICHLLS 2L system compared to 300 z-galvo positions of the original LLS or IHLLS 1L.

In the present configuration single image frames can be obtained in between 50 and 100 ms. This will allow complete volumes of up to 100 μm^2^ × 100 μm^2^ × 80 μm with 9 z-galvo positions and 4 phase shifts to be obtained in as little as 1.8 s in single colors. With smaller volumes these rates can be improved even further. To image multiple colors adds complexity. Bandpass filters with multiple transmission notches can be used. However, emission bands must be narrow to prevent blurring of the hologram. If bands are too close in wavelength, then bleed-through from shorter wavelengths to longer is an issue. This can be prevented by sufficiently separated wavelengths. On our system with excitation lasers at 405, 488,561 and 642 nm, emission bands can be combined with centers at 520 and 660 nm and at 420 and 580 nm to ensure no bleed-through. If these two multiple bandpass filters are combined with two cameras, then switching of emission filters can be eliminated, and the Bessell beam excitation and emission SLMs can be coordinated allowing two color emission to be captured by a simple doubling of the time for each volume. Alternatively, a color camera with specific bandpass filters over the photosensitive elements would allow single camera detection. Another solution is to use a mechanical filter switcher with just one camera and the dual bandpass approach described above. Filter switching with a fast device would slow acquisition by about 100 ms for each two-color changes, once per volume acquisition.

Our goal is to enable automated and repetitive capture of similar volumes over time to achieve spatial and temporal resolutions to track dynamic movements of cellular structure in 3D over time. However, to enable very high-resolution phase and amplitude images we will need to automate 3D scanning and ICHLLS 2L imaging in up to all 4 colors of the original LLS system by sweeping excitation through hundreds of *z*-axis planes. Our approach will enable high temporal resolution of the spatial relationships between synaptic and cellular structures and retain both amplitude and phase information in the reconstructed images. Understanding colocalization of different labeled proteins and cellular elements and their interaction is crucial to understanding brain function and the low light excitation of LLS coupled with enhanced sensitivity of ICHLLS-2L will enable this.

## Supplementary Material

Presentation 1

## Figures and Tables

**TABLE 1:** Quantitative analysis of colocalization using all three imaging techniques, IHLLS 1L and IHLLS 2L reconstructed amplitude and phase, shown for the neuron in Figure 4.

Technique	Pearson’s PCC	Manders’ MOC	Manders’ M1	Manders’ M2
IHLLS 1L	0.941	0.9328	0.973	0.972
IHLLS 2L Amplitude	0.93	0.9306	1	0.992
IHLLS 2L Phase	0.712	0.7249	0.8297	0.846

## Data Availability

The original contributions presented in the study are included in the article/Supplementary Material, further inquiries can be directed to the corresponding author.
