# Supplementary material for: Incoherent color holography lattice light-sheet for subcellular imaging of dynamic structures
Source: Front Photon. Author manuscript; Available in PMC 2025 Feb 5. (PMC11798417; doi:10.3389/fphot.2023.1096294)
Supplement: Presentation 1 [file NIHMS2046459-supplement-Presentation_1.pdf]

Supplementary Material

Supplementary Figure

Optical simulations of ICHLLS were performed in two steps for each of the excitation wavelengths, λ_1 = 488 nm or λ_2 = 561 nm. First, the correct distances between each sequential optical component and the focal length of f_{SLM} of a diffractive lens superimposed on the SLM, were calculated to match the transversal pixel magnification of 62.5 in both configurations, conventional LLS, and ICHLLS 1L, using OpticStudio (Zemax, LLC) optical design, Figure (S1 a). It was determined that for an emission wavelength of 520 nm, and with an overall transversal magnification set to 62.5, the distances should be about, $d_1 = 75\ m$m , $d_2 = 95.074\ mm$, $d_3 = 288.914\ mm$, $d_4 = 103.660\ mm$, $d_5 = 103.660\ mm$, $d_6 = 288.914\ mm$, and the distance between the lens TL_4 to the detector should be about 664 mm (i.e., $d_7 = 664\ mm$) , and the focal length of the single diffractive lens superimposed on the SLM was determined to $\mathrm{f_{SLM,488\ nm}}$ = 400 mm or $\mathrm{f_{SLM,561\ nm}}$ = 415 mm. For this step, the transversal magnification of 62.5 was checked by imaging the USAF 1951 resolution target. Second, an optimization of a multi-configuration optical system, Figure (S1 b, c), were performed to calculate the focal lengths of the two diffractive lenses superimposed on the SLM for each excitation wavelength to provide maximum overlap of the two beams at the plane of the detector but keeping fixed all the distances $d_1 \div d_7$.found in the previous step. After performing the optimization, the values of the two focal lengths were found to $\mathrm{f_{d1,488nm}} = 220$ mm , $\mathrm{f_{d2,488nm}} = 2356$ mm , $\mathrm{f_{d1,561nm}} = 228$ mm , and $\mathrm{f_{d2,561nm}} = 2444$ m., which were used for the design of the two diffractive lenses. These two lenses focus on a distance $d_{7\_1} = 555.185\,mm$ in the front of the camera, Figure (S1 b), and at $d_{7\_1} = 826.793\,mm$ behind the camera, Figure (S1 c), respectively. In implementation, the distance $d_{7\_1} + d_8$ may need to be tuned by ±0.3 depending on tolerances and imperfections of optical parameters of other elements of the system (e.g., tolerances of lenses, tolerances of phase resolution of the SLM, etc.). (i.e., $d_{7\_1} + d_8 = 664.298\,mm$, when calculating f_{d1} , and $d_{7\_1} + d_8 = 663.793\,mm$, when calculating f_{d2}.

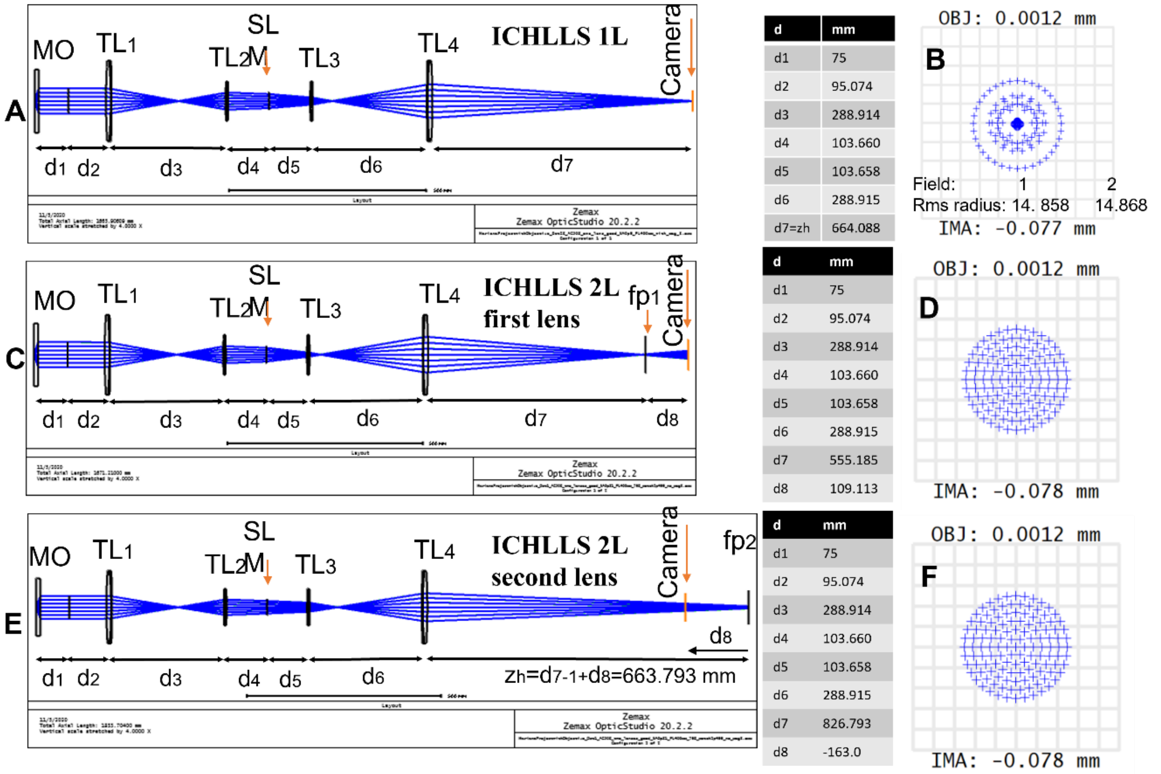

d	mm
d1	75
d2	95.074
d3	288.914
d4	103.660
d5	103.658
d6	288.915
d7=zh	664.088

d	mm
d1	75
d2	95.074
d3	288.914
d4	103.660
d5	103.658
d6	288.915
d7	555.185
d8	109.113

d	mm
d1	75
d2	95.074
d3	288.914
d4	103.660
d5	103.658
d6	288.915
d7	826.793
d8	-163.0

Supplementary Figure S1. Optical design of the ICHLLS; (A) Ray tracing using only one diffractive lens; (B) The spot diagram from (A); (C) Ray tracing using two diffractive lenses, f_{p1} the first focus position; (D) The spot diagram from (C); (E) Ray tracing using two diffractive lenses, f_{p2} the second focus position; (F) The spot diagram from (E). The distance d_1 is the distance between the MO and a dummy surface with infinite radius needed to check the beam collimation after the MO. The distance d_2 is the distance between the dummy surface and lens TL_1.
